# Neurophysiological Differences Between Women With Fibromyalgia and Healthy Controls During Dual Task: A Pilot Study

**DOI:** 10.3389/fpsyg.2020.558849

**Published:** 2020-11-04

**Authors:** Santos Villafaina, Juan Pedro Fuentes-García, Ricardo Cano-Plasencia, Narcis Gusi

**Affiliations:** ^1^Physical Activity and Quality of Life Research Group (AFYCAV), Faculty of Sport Sciences, University of Extremadura, Cáceres, Spain; ^2^Faculty of Sport Sciences, University of Extremadura, Cáceres, Spain; ^3^Clinical Neurophysiology, San Pedro de Alcántara Hospital, Cáceres, Spain

**Keywords:** dual task, pain, physical fitness, EEG, strength

## Abstract

**Background:**

Women with FM have a reduced ability to perform two simultaneous tasks. However, the impact of dual task (DT) on the neurophysiological response of women with FM has not been studied.

**Objective:**

To explore both the neurophysiological response and physical performance of women with FM and healthy controls while performing a DT (motor–cognitive).

**Design:**

Cross-sectional study.

**Methods:**

A total of 17 women with FM and 19 age- and sex-matched healthy controls (1:1 ratio) were recruited. The electroencephalographic (EEG) activity was recorded while participants performed two simultaneous tasks: a motor (30 seconds arm-curl test) and a cognitive (remembering three unrelated words). Theta (4–7 Hz), alpha (8–12 Hz), and beta (13–30) frequency bands were analyzed by using EEGLAB.

**Results:**

Significant differences were obtained in the healthy control group between single task (ST) and DT in the theta, alpha, and beta frequency bands (*p*-value < 0.05). Neurophysiological differences between ST and DT were not found in women with FM. In addition, between-group differences were found in the alpha and beta frequency bands between healthy and FM groups, with lower values of beta and alpha in the FM group. Therefore, significant group^∗^condition interactions were detected in the alpha and beta frequency bands. Regarding physical condition performance, between groups, analyses showed that women with FM obtained significantly worse results in the arm curl test than healthy controls, in both ST and DT.

**Conclusion:**

Women with FM showed the same electrical brain activity pattern during ST and DT conditions, whereas healthy controls seem to adapt their brain activity to task commitment. This is the first study that investigates the neurophysiological response of women with FM while simultaneously performing a motor and a cognitive task.

## Introduction

Fibromyalgia (FM) is characterized by chronic, widespread, and persistent pain and its prevalence is around 2% to 3% worldwide (Helmick et al., 2008). Nevertheless, FM is accompanied by other symptoms, such as stiffness, mobility problems, sleep disorders, anxiety, depression, or cognitive impairments (Wolfe et al., 2010). The study of cognition and FM is a growing field of research since previous studies have reported cognitive impairments in several domains of people with FM, including long-term memory (Canovas et al., 2009), short-term memory (Park et al., 2001), working memory (Coppieters et al., 2015), processing speed (Del Paso et al., 2015), or even an abnormal EEG signal at rest (Villafaina et al., 2019a,b).

Due to the impact of FM symptoms, people with FM showed a diminished quality of life and reduced ability to perform activities of daily living (Burckhardt et al., 1993; Huijnen et al., 2015). In this regard, activities of daily living are commonly presented under the dual-task paradigm (where two or even more tasks are simultaneously required) (Yuan et al., 2015). People with FM have shown a diminished physical performance when two tasks are simultaneously presented during postural control (de Gier et al., 2003), balance (Villafaina et al., 2019c) or when compared with healthy controls in physical fitness tests (Villafaina et al., 2018, 2019d).

Progress in mobile and wireless technologies have allowed studying the impact of DT on the electroencephalography (EEG) (Wagner et al., 2012; Winslow et al., 2016) during ecological scenarios. In this regard, previous studies have explored brain dynamics during dual-task conditions in young and older people (De Sanctis et al., 2014; Malcolm et al., 2015; Bogost et al., 2016). However, the impact of DT on neurophysiological measures has not been investigated. Therefore, the present study aimed to explore the neurophysiological response of women with FM and healthy controls while performing a DT (motor–cognitive). We hypothesized that DT would engage brain areas related to executive function (prefrontal cortex) (Ford et al., 2002; Giraud et al., 2007) and areas related to sensorimotor integration (sensorimotor cortex and posterior parietal cortex) (Sipp et al., 2013; Beurskens et al., 2016; Bradford et al., 2016). Moreover, neurophysiological differences between healthy controls and people with FM could emerge since people with FM reported an attention deficit disorder (van Rensburg et al., 2018; Yilmaz and Tamam, 2018). This is because people with FM have impaired the ability to perform two simultaneous tasks (Huijnen et al., 2015; Villafaina et al., 2018, 2019d).

## Materials and Methods

### Participants

Twenty-five women with fibromyalgia participated in this study with a cross-sectional design. They were divided into two groups: (1) women with fibromyalgia (*N* = 17; age = 51.88 [7.30]) and (2) age- and gender-matched healthy controls (*N* = 19; age = 50.95 [6.83]) (Table 1). The Extremadura Association of Fibromyalgia (Spain) recruited the women with FM by telephone calls in April 2018.

**TABLE 1:** Participants’ characteristics.

Measurements	Women with FM Mean (SD)	Healthy controls Mean (SD)	*p*-value
Sample size (*N*)	17	19	
Age (years)	51.88 (7.30)	50.95 (6.83)	0.975
BMI (kg/m^2^)	27.54 (4.69)	24.07 (3.89)	0.029
Fat mass (%)	26.77 (7.85)	19.34 (7.33)	0.010
VAS for pain (0–100)	63.53 (16.18)	−	−
FIQ total score	53.31 (10.29)	−	−

*BMI, body mass index; FIQ, fibromyalgia impact questionnaire; VAS, Visual Analog Scale.*

The inclusion criteria for participants were as follows: (a) be a female and aged between 30 and 75 years, (b) be able to communicate with technicians, and (c) have read and signed the written informed consent. In addition, women with FM have to be diagnosed by a rheumatologist, according to the American College of Rheumatology’s criteria (Wolfe et al., 2010). However, the following exclusion criteria were defined as follows: (a) had contraindications for physical activity, (b) have been suffering from a psychiatric or neurological diagnoses according to their current medical history, and (c) were pregnant. Moreover, healthy controls were excluded if they suffered from any pain that lasted for more than three months in the last six months.

The University of Extremadura bioethical research committee approved the procedures (approval number: 62/2017), following the updated Declaration of Helsinki. All the participants read and signed the informed consent prior to the first assessment.

### Procedure

The body composition measurement was measured using the using the Tanita Body Composition Analyzer BC-418 MA. Moreover, the impact of the disease was evaluated with the (Bennett, 2005; Esteve-Vives et al., 2007) Spanish version of the Fibromyalgia Impact Questionnaire (FIQ), which evaluates the impact of symptoms (in several domains such as pain, fatigue, rested, stiffness, anxiety, depression, physical impairment, feeling good, or work missed). Furthermore, the pain intensity was measured through the VAS for pain (0–100) (Boonstra et al., 2008; Hawker et al., 2011), referring to the day they were evaluated. Body composition was evaluated in both FM and healthy groups whereas the impact of the disease and pain intensity were assessed (through an interview) exclusively in the FM group.

EEG was recorded while participants performed the arm-curl test (Ariadna Aparicio et al., 2015). Therefore, participants have to be seated in a chair holding a 2.3-kg weight and encouraged to perform, as many times as possible for 30 s, arm curls (to lift the weight and return to the starting position) through a full range of motion. This physical fitness test was selected since it could potentially discriminate women with fibromyalgia from healthy women (Ariadna Aparicio et al., 2015) and the performance is associated with the severity of the symptoms (Soriano-Maldonado et al., 2015). The best of the two trials, for each arm, was chosen for analyses purposed.

Participants performed the physical fitness test in both single-task (ST) and DT conditions. The simultaneous cognitive task consisted of remembering three random words. Therefore, participants were encouraged to think in these words while the arm-curl test was being performed. The cognitive performance and correct responses were registered. Conditions (ST and DT) were randomized.

### Instrument and Measures

The EEG signal was record using the Enobio device (Neuroelectrics, Cambridge, MA, United States) (Ruffini et al., 2007). The EEG signal was recorded in a total of 19 scalp locations according to the International 10–20 system (Homan, 1988) in different brain areas such as frontal (Fz, Fp1, Fp2, F3, F4, F7, and F8), central (Cz, C3, and C4), temporal (T3, T4, T5, and T6), parietal (Pz, P3, and P4), and occipital (O1 and O2).

Two electrodes placed in each mastoid served as reference. Moreover, the impedance was kept below 5 KΩ and a sampling rate of 500 Hz was used. Preprocessing steps and data analyses were conducted with the EEGlab toolbox (MatLab) (Delorme and Makeig, 2004). A 1-Hz high-pass filter was used, and the line noise was removed using the CleanLine algorithm in EEGlab. In order to reject bad channels and correct continuous data, the Artifact Subspace Reconstruction (ASR) was used. In this regard, if a channel is correlated with the surrounding channels less than 0.8, the channel was rejected. Moreover, principal components (PCs) were classified into high variance (in this case, with a standard deviation of 8 from the calibration data) or normal variance. A window rejection criterion of 0.25 was set, meaning that if more than 0.25 of channels are judged to be bad even after ASR, the window will be rejected. Then, bad channels were interpolated and the data was re-referenced to average. In addition, the independent component analysis (ICA) was conducted (Jung et al., 2000) and single equivalent current dipoles estimated, looking for the symmetrically constrained bilateral dipoles. Dipoles located outside the brain were removed using the independent components (ICs) when the dipoles’ residual variance was larger than 15%. Moreover, a visual inspection of dipoles located inside or outside the brain was performed. Lastly, the fast Fourier transform (FFT) method was used to compute spectral decomposition after splitting continuous data in 1-s epochs. Therefore, theta (4–7 Hz), alpha (8–12), and beta (13–30) power spectrums were computed.

Additionally, in order to report EEG sources analysis, evoke-related potentials (ERPs) were generated with a 7000-ms window time-locked, and dipoles were then estimated utilizing DIPFIT. Thus, a Kmeans cluster procedure (*k* = 10) was performed for clustering dipoles, using dipole location. Different clusters were performed according to the group and condition. Thus, for within- and between-group analysis purposes, clusters were obtained taking into account their group (fibromyalgia or healthy control) and condition (dual or single task). Therefore, ERSP were extracted to observe the amplitude and latency as well as the time–frequency analysis for source analyses. For analysis purposes, only clusters which contain the signal of the majority of the sample were selected. Results from these analyses are presented in Supplementary File 1.

### Statistical Analysis

A 2 × 2 design using the EEGLAB study design was used to explore the EEG data during ST versus DT in both healthy and women with FM. Permutation statistics with 2000 repetitions and the false discovery rate (FDR) correction were applied for EEG analyses.

In addition, the SPSS statistical package (version 22.0; SPSS, Inc., Chicago, IL, United States) was used to analyze the arm-curl test performance in both ST and DT conditions. Moreover, non-parametric analyses were conducted taking into account the results of Shapiro–Wilk and Kolmogorov–Smirnov tests. Therefore, the Wilcoxon signed-rank test was used to assess differences within groups, whereas Mann–Whitney U or chi-squared tests (when appropriate) were used to explore between-group differences in both ST and DT conditions. Additionally, the dual-task cost (DTC), which measures the losses of performance due to motor–cognitive interference, was calculated as follows:

*DTC* = *(Result of DT condition – Result of ST condition)/Result of ST condition.*

Effect size [*r*] was calculated for each the ST and DT comparisons (Fritz et al., 2012). Values of 0.37, 0.24, and 0.10 represent large, medium, and small effect sizes, respectively (McGrath and Meyer, 2006). The alpha level of significance (0.05) was adjusted according to the Benjamini–Hochberg procedure to avoid type I error derived from multiple comparisons (Benjamini and Hochberg, 1995).

## Results

### Impacts of Dual Task on Physical and Cognitive Performance

Table 2 shows within- and between-group differences. Regarding within group analyses, differences were not found in women with FM nor healthy controls (*p*-value > 0.05). Nevertheless, between groups, analyses showed that women with FM obtained significantly worse results in the arm curl test than healthy controls in both DT (*p*-value < 0.001) and ST (*p*-value < 0.001) conditions.

**TABLE 2:** Within- and between-group comparisons in the arm-curl test during ST and DT in women with FM and healthy controls.

Arm-curl performance (rep)	ST Median (IQR)	DT Median (IQR)	*Z*	*p*-value	Effect size
*Within-group comparisons*					
Women with FM	17.00 (4.5)	15.50 (4.3)	−1.082	0.279	0.262
Healthy controls	23.50 (3.5)	23.00 (4.5)	−0.260	0.795	0.248
*Between-group comparisons*					
*Z*	−3.966	−4.488			
*p*-value	< 0.001	<0.001			
Effect size	−0.661	−0.748			

*DT, dual task; DTC, dual-task cost; IQR, interquartile range; Rep, repetitions; ST, single task. *p*-value < 0.05.*

Table 3 shows the between-group differences in the arm-curl test DTC and cognitive performance (successful responses). Differences between healthy controls and women with FM were not observed in the DTC.

**TABLE 3:** Between-group comparisons in the dual-task cost and cognitive performance in the arm-curl test in women with FM and healthy controls.

Arm-curl performance (rep)	Women with FM Median (IQR)	Healthy controls Median (IQR)	*Z*	*p*-value	Effect size
Successful responses	3 (0)	3 (0)	−	0.615^a^	−
Dual-task cost	−0.04 (0.17)	0.04 (0.12)	−1.016	0.315^b^	0.170

*DT, dual task; DTC, dual-task cost; IQR, interquartile range; Rep, repetitions; ST, single task. ^*a*^*p*-value obtained from chi-squared test. ^*b*^*p*-value obtained from Mann–Whitney U test. *p*-value < 0.05.*

### Impacts of Dual Task on EEG Frequency Bands

**FIGURE 1:**
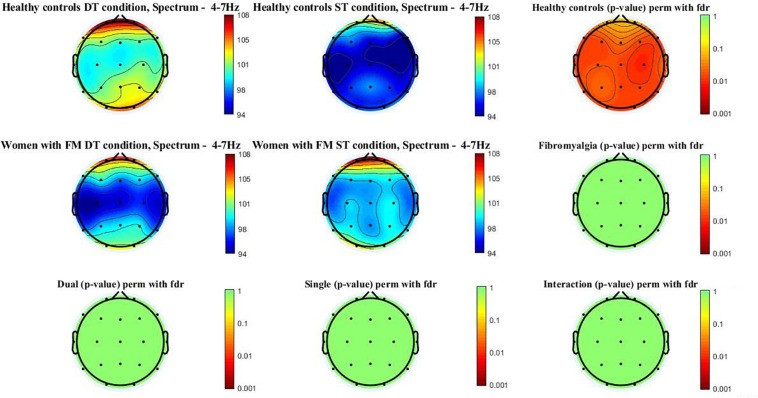
Theta power spectrum (4–7 Hz) topographic maps in women with FM and healthy controls. Differences were only located (*p* < 0.05) between ST and DT conditions in healthy controls in the following scalp locations: frontal (Fz, F3, F4, F7, and F8), central (Cz, C3, and C4), temporal (T3, T4, T5, and T6), parietal (Pz, P3, and P4), and occipital (O1 and O2).

Figure 1 shows the theta power spectrum (4–7 Hz) topographic maps in the women with FM and healthy controls during ST and DT conditions. Differences (*p*-value < 0.05) were only observed in healthy controls when compared ST and DT conditions. Significant differences between groups (*p*-value < 0.005) were located in the frontal (Fz, F3, F4, F7, and F8), central (Cz, C3, and C4), temporal (T3, T4, T5, and T6), parietal (Pz, P3, and P4), and occipital (O1 and O2). Higher theta power spectrum values were found in the DT compared to ST values. However, significant between-group differences or group^∗^condition interactions were not found.

**FIGURE 2:**
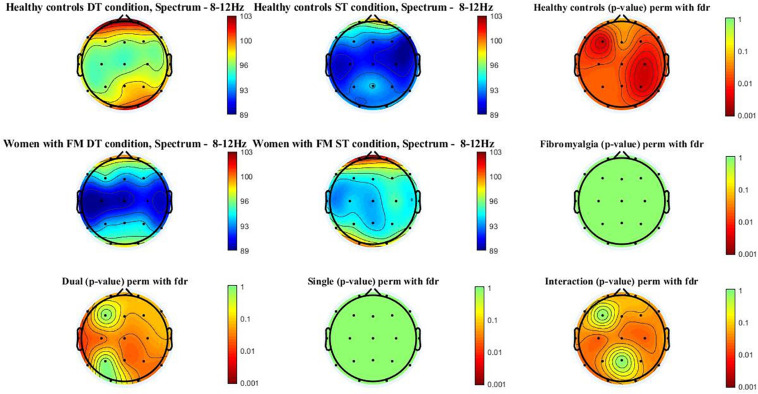
Alpha power spectrum (8–12 Hz) topographic maps in women with FM and healthy controls. Differences were only located (*p* < 0.05) between ST and DT conditions in healthy controls and between healthy controls and women with FM in the DT condition. In addition, the interactions group*condition is significant in the following scalp locations: central (Cz, C3, and C4), temporal (T5 and T6), and occipital (O1).

Figure 2 shows the alpha power spectrum (8–12 Hz) topographic maps in the women with FM and healthy controls during ST and DT conditions. Differences (*p*-value < 0.05) were observed in healthy control when compared ST and DT conditions [scalp locations: frontal (Fz, Fp1, Fp2, F3, F4, F7, and F8), central (Cz, C3, and C4), temporal (T3, T4, T5, and T6), parietal (Pz, P3, and P4), and occipital (O1 and O2)]. Moreover, significant between-group differences (p-value < 0.005) were found when comparing healthy controls and women with FM in the DT condition [scalp locations: frontal (F7), central (Cz, C3, and C4), temporal (T3, T5, and T6), parietal (Pz and P4), and occipital (O2)]. The group^∗^condition interactions were also significant in the frontal (Fp1, F4, F7, and F8), central (Cz, C3, and C4), temporal (T3, T5, and T6), and occipital (O1).

**FIGURE 3:**
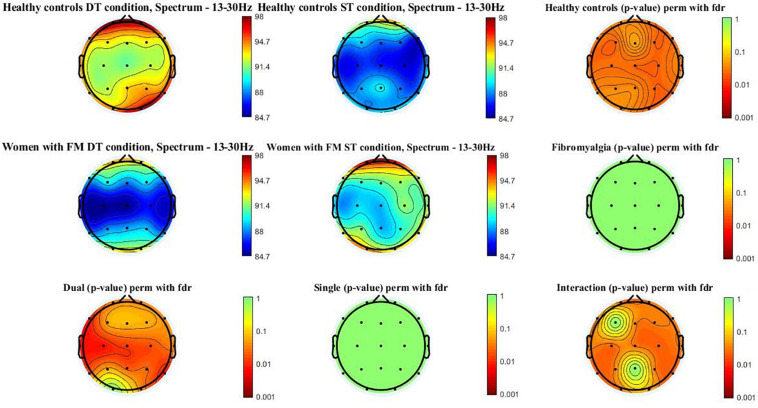
Beta power spectrum (13–30 Hz) topographic maps in women with FM and healthy controls. Differences were only located (*p* < 0.05) between ST and DT conditions in healthy controls and between healthy controls and women with FM in the DT condition. In addition, the interactions group*condition is significant in the following scalp locations: frontal (Fp1, Fp2, Fz, F4, F7, and F8), central (Cz, C3, and C4), temporal (T3, T4, T5, and T6), parietal (P3 and P4), and occipital (O1).

Figure 3 shows the beta power spectrum (13–30 Hz) topographic maps in the women with FM and healthy controls during ST and DT conditions. Differences (*p*-value < 0.05) were observed in healthy control when comparing ST and DT conditions [scalp locations: frontal (Fz, Fp1, Fp2, F3, F4, F7, and F8), central (Cz, C3, and C4), temporal (T3, T4, T5, and T6), parietal (Pz, P3, and P4), and occipital (O1 and O2)]. Moreover, significant between-group differences (*p*-value < 0.005) were found when comparing healthy controls and women with FM in the DT condition [scalp locations: frontal (F7, F8, Fp1, and Fp2), central (Cz, C3, and C4), temporal (T3, T4, T5, and T6), parietal (Pz and P4), and occipital (O2)]. The group^∗^condition interactions were also significant in the frontal (Fp1, Fp2, Fz, F4, F7, and F8), central (Cz, C3, and C4), temporal (T3, T4, T5, and T6), parietal (P3 and P4), and occipital (O1).

### Source Analyses

Supplementary File 1 shows the results of the complementary ERSP sources analyses. Differences within-group (ST vs. DT) were found in both healthy and FM group in one cluster for each group. In the healthy control group, the centroid dipole was located in the left medial frontal gyrus (Talairach coordinates: *X* = −4, *Y* = 56, *Z* = −5), whereas in the fibromyalgia group, the centroid dipole was located in the right middle temporal gyrus (Talairach coordinates: *X* = 67, *Y* = −23, *Z* = −5).

Between-group differences (fibromyalgia vs. healthy control group) showed differences during dual- and single-task conditions. During dual-task conditions, the centroid dipoles were located in the right cuneus (Talairach coordinates: *X* = 11, *Y* = −73, *Z* = 15) and right medial frontal gyrus (Talairach coordinates: *X* = 1, *Y* = 58, *Z* = −12). During single-task conditions, the centroid dipole was located in the right medial frontal gyrus (Talairach coordinates: *X* = 8, *Y* = 48, *Z* = 7).

## Discussion

The main purpose of this study was to explore the neurophysiological response of women with FM and healthy controls while performing a DT (motor–cognitive). Significant differences were obtained in the healthy control group between ST and DT in the theta, alpha, and beta frequency bands. Nevertheless, significant within-group differences were not obtained in women with FM between ST and DT in any of the frequency bands. In addition, between-group differences were found in the alpha and beta frequency bands between healthy and FM groups, with lower values of beta and alpha in the FM group. Therefore, significant group^∗^condition interactions were detected in the alpha and beta frequency bands. Regarding physical condition performance, between groups, analyses showed that women with FM obtained significantly worse results in the arm curl test than healthy controls, in both ST and DT.

Previous studies have reported differences between healthy controls and women with FM in the EEG signal at rest (Fallon et al., 2017; Villafaina et al., 2019a). However, this is the first study reporting differences between healthy controls and women with FM during DT conditions. Previous investigations have explored the brain activity while dual-tasking. Most of these studies have used the functional near-infrared spectroscopy (fNIRS) (Holtzer et al., 2011, 2015; Al-Yahya et al., 2016; Lin and Lin, 2016; Maidan et al., 2017) over the prefrontal cortex to investigate this topic. In this regard, increases in oxygen levels in the prefrontal cortex have been reported when performed any type of DT (Holtzer et al., 2015; Al-Yahya et al., 2016; Lin and Lin, 2016). However, results from fNIRS studies are limited to the prefrontal area due to the restricted number of channels that could be recorded. Thus, previous studies have employed the EEG to study the impact of DT on brain activity (De Sanctis et al., 2014; Malcolm et al., 2015; Bogost et al., 2016). Our results are consistent with a previous study where an increase in theta and beta power spectrum were observed between ST and DT (Pizzamiglio et al., 2017) in the frontal and parietal regions in healthy adults.

However, in the study of Pizzamiglio et al. (2017), a decrease in the alpha power spectrum during the DT was detected when compared with the ST condition. This is inconsistent with our results, where a significant increase in the DT was found in the healthy control group when compared with the ST condition. However, hypothetically, it could be due to the type of DT, which is presented in this study (a memory-based DT, which consisted of remembering three random unrelated words while the women were performing the tests). In this regard, findings of alpha power have not been consistent across experimental studies (Wianda and Ross, 2019). In our study, participants were encouraged to keep in mind these unrelated words while performing the motor task. This could be connected with the findings of a previous study where an alpha increase during the retention interval in a short-term memory task was reported (Jensen et al., 2002). Interestingly, another study has suggested that the increase in alpha power spectrum plays an active role in preventing the distracting information into areas which retain the memory items (Mazaheri et al., 2014). Moreover, previous studies have linked beta band to short-term memory (Tallon-Baudry et al., 1999; Palva et al., 2011), elevated mental workload (Coelli et al., 2015), or concentration (Kakkos et al., 2019) as well as increasing in working memory (Spitzer and Haegens, 2017). In the same line, the theta band is associated to increases in cognitive workload (Fuentes-García et al., 2019; Diaz-Piedra et al., 2020). Therefore, further investigation is needed to clarify the role of alpha, beta, and theta bands in different types of DT conditions.

The arm curl provides useful information in people with fibromyalgia since it could potentially be used to discriminate (in ST conditions) women with fibromyalgia from healthy women (Ariadna Aparicio et al., 2015) or even the severity of the symptoms (Soriano-Maldonado et al., 2015). Our results showed differences between women with FM and healthy controls in both ST and DT in this physical fitness test. Nevertheless, within-group differences were not observed in women with FM nor healthy controls in the ST or DT conditions. These results are consistent with previous investigations where similar results using both the arm-curl test and the same simultaneous cognitive task were observed (Villafaina et al., 2019d). These results, as previously suggested by Villafaina et al. (2018), could be due to a low complexity of the motor task. However, since the arm curl test has not been deeply studied under DT conditions in women with FM, further studies are necessary to confirm this hypothesis.

However, neurophysiological results showed that healthy controls modified their brain activity between ST and DT in order to adapt their brain activity to the task commitment. These changes were not observed in the women with FM between the ST and the DT conditions. This could be derived from the attention deficit disorder (van Rensburg et al., 2018; Yilmaz and Tamam, 2018), which is common in this population. Besides, people with FM usually showed depression or cognitive impairment (Wolfe et al., 2010) in several domains such as long-term memory (Canovas et al., 2009), short-term memory (Park et al., 2001), or working memory (Coppieters et al., 2015). These comorbidities could have an impact on EEG, showing a left hemisphere hypoactivation (Villafaina et al., 2019e) or a reduced theta power at rest (Musaeus et al., 2018; Villafaina et al., 2019b). However, taking into account the simultaneous cognitive task, the attention deficit disorder could have a significant impact on the EEG patter during the DT condition. The attention deficit disorder could lead to difficulties in focusing their attention on three unrelated words provided before the test started, “forgetting” to think in these words during the DT condition. This, hypothetically, may explain that changes in the neurophysiological measures were not found. For instance, it could be expected, as occurred in the healthy group, that beta and theta bands would increase during the DT condition due to higher cognitive demands or workload (Fuentes-García et al., 2019; Diaz-Piedra et al., 2020). This is because increases in beta and theta bands, due to increases in cognitive workload, could be expected. Thus, EEG measurement could be an interesting tool to enhance the knowledge about the DT paradigm as well as help to interpret the reasons behind differences between healthy controls and women with FM in the physical performance in both DT and ST conditions.

Source analysis shows differences within group (ST vs. DT) in both healthy and FM groups. In the healthy control group, the centroid dipole was located in the right anterior cingulate, whereas in the fibromyalgia group, the centroid dipole was located in the left cingulate gyrus. In this regard, source analysis has been used to investigate the electrocortical source during ST and DT paradigms (Lin et al., 2011; Bogost et al., 2016). A previous study showed a reduction in the mean absolute N1 ERP peak amplitude in the DT compared with the ST in the N1 between ST and DT conditions (Bogost et al., 2016). However, the dual-task employed in this investigation (visual working memory) does not allow to compare the results. Thus, further investigation in this field is required.

This study has some limitations. First, all the participants were women, so results cannot be extrapolated to men with FM. In the same line, the relatively small sample size (17 women with FM and 19 healthy controls) could make that only greater differences would reach the significance level. Thus, results cannot be generalized and further research in this field is necessary. Third, the lack of test–retest reliability, validity, and variability of the EEG results should be considered. Therefore, an EEG register longer than 30 s should be recommended as well as the inclusion of a baseline assessment. Lastly, according to their current medical history any psychiatric or neurological diagnoses were present in their medical history. However, due to the high rate of comorbidities with several psychiatric disorders in FM (such as mood, anxiety, or depression) (Sancassiani et al., 2017) could not be discarded that some of the participants might be suffering from these disorders when women with FM were assessed. Taking into account these limitations, results should be taken with caution.

## Conclusion

Neurophysiological differences between women with FM and healthy controls were found during DT condition. This is the first study which investigates the neurophysiological response of women with FM while simultaneously performing a motor and a cognitive task. Women with FM showed the same brain activity pattern during ST and DT conditions, whereas healthy controls seem to adapt their brain activity to task commitment.

## Data Availability Statement

The raw data supporting the conclusions of this article will be made available by the authors, without undue reservation.

## Ethics Statement

The studies involving human participants were reviewed and approved by the University of Extremadura Bioethical Committee. The patients/participants provided their written informed consent to participate in this study.

## Author Contributions

JF-G, SV, and NG conceived the study. SV and RC-P collected the data. SV and RC-P analyzed the data. JF-G, NG, and SV designed the figures and tables. SV, JF-G, and NG wrote the manuscript. NG, SV, JF-G, and RC-P provided critical revisions on the successive drafts. All authors approved the manuscript in its final form.

## Conflict of Interest

The authors declare that the research was conducted in the absence of any commercial or financial relationships that could be construed as a potential conflict of interest.
