# Supplementary material for: Neurophysiological Differences Between Women With Fibromyalgia and Healthy Controls During Dual Task: A Pilot Study
Source: Front Psychol. 2020 Nov 4;11:558849. doi: 10.3389/fpsyg.2020.558849 (PMC7672184; doi:10.3389/fpsyg.2020.558849)
Supplement: Supplementary file 1 [file Data_Sheet_1.docx]

**SOURCES ANALYSIS**

Table 1 shows the ERPs source analyses results by clustering dipoles, reporting cluster number, location and anatomical area when compared single vs dual task for each group (fibromyalgia and healthy control). Results from ERSP sources analyses showed two clusters where significant differences were found.

**Table S1.** Comparison between single and dual tasks source analyses in both healthy and fibromyalgia groups.

| Cluster number | Location  (Tailarach coordinates) | Anatomical area |
| --- | --- | --- |
| Healthy control group | | |
| 2* | X= -4, Y=56, Z= -5 | L Medial frontal gyrus |
| 8 | X= -50, Y= -40, Z= -7 | L Sub-Gyral |
| 9 | X= 28, Y= -50, Z= 45 | R Precuneus |
| 10 | X= 60, Y=-7, Z= -6 | R Middle temporal gyrus |
| Fibromyalgia group | | |
| 2* | X= 67, Y= -23, Z= -5 | R Middle temporal gyrus |
| 6 | X= -7, Y= 52, Z= -10 | L Medial frontal gyrus |
| 8 | X= -63, Y= -11, Z= 4 | L Superior temporal gyrus |
| 9 | X= 7, Y= -78, Z= 12 | R Cuneus |
| 10 | X= 36, Y= 16, Z= 49 | R Middle frontal gyrus |
| * Significant differences (p-vale<0.05) were found in the ERSP.  R: Right; L: Left | | |
|  | | |

As shown in Table S1 a significant difference was found in the ERSP, mainly located between 5-9 Hz (see Figure S1) in the control group. The centroid dipole was located in the left medial frontal gyrus (Talairach coordinates X= -4, Y=56, Z= -5) (Figure S1).


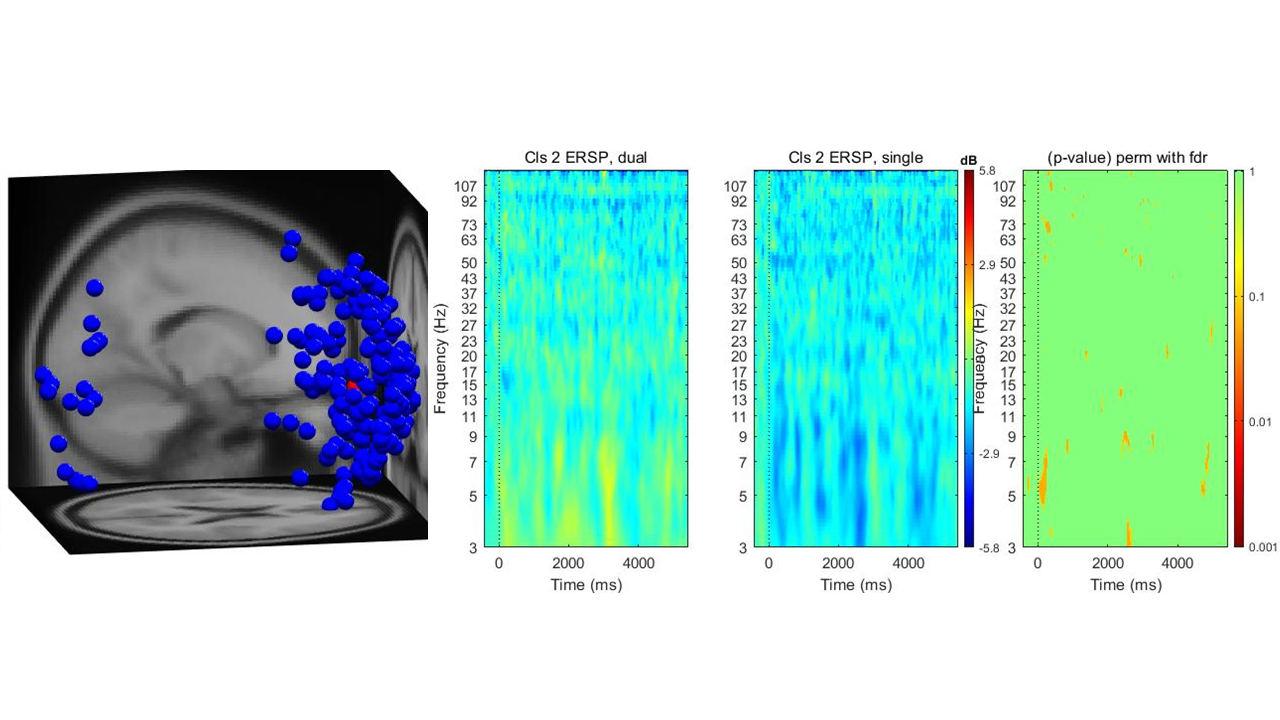


**Figure S1.** Dipoles location and ERSP of a cluster of dipoles when compared single and dual-task conditions in the healthy group.

Moreover, in the fibromyalgia group, a statistically significant difference was found in the ERPS, mainly located between 5-9 Hz (see Figure S2) when compared single and dual task conditions. The centroid dipole was located in the right medial frontal gyrus (Talairach coordinates X= 67, Y= -23, Z= -5).


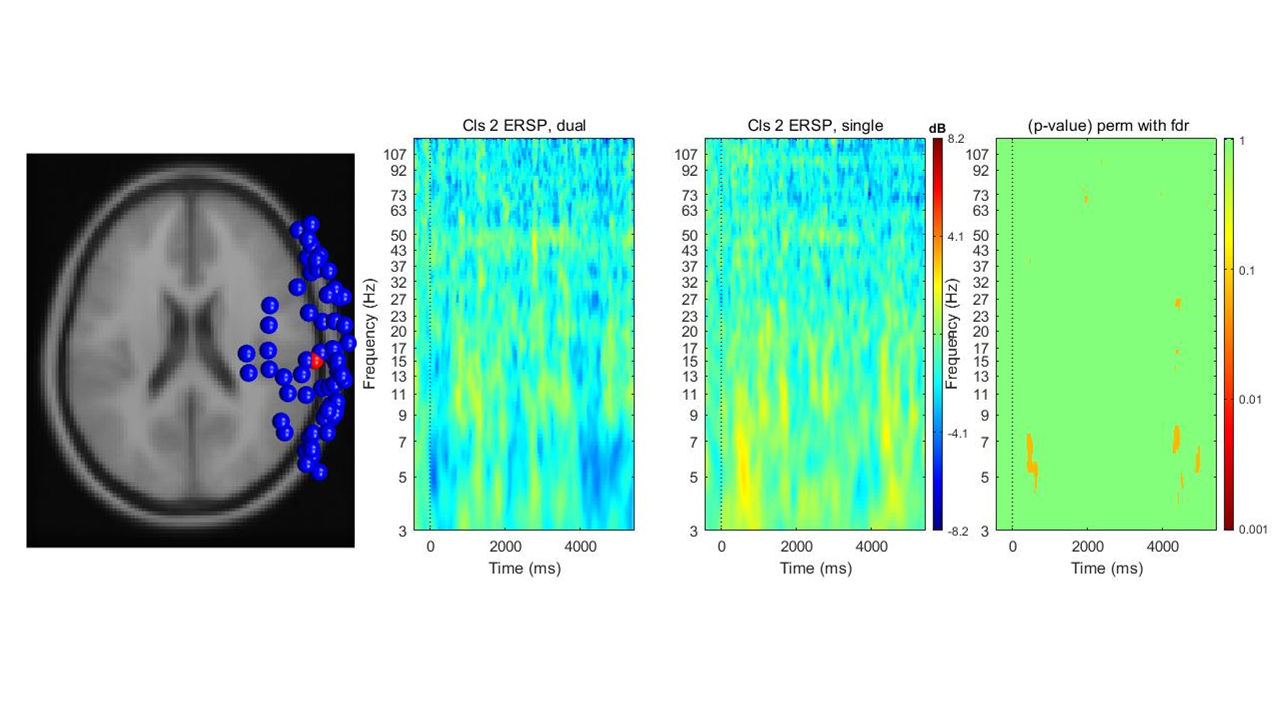


**Figure S2.** Dipoles location and ERSP of a cluster of dipoles when compared single and dual-task conditions in the fibromyalgia group.

Table S2 shows the ERSP source analyses results by clustering dipoles, reporting cluster number, location and anatomical area when compared fibromyalgia vs healthy control group for each task condition (dual and single tasks). Results from ERSP sources analyses show significant differences between fibromyalgia and healthy control group in two clusters (see Figure S3 and Figure S4). Furthermore, one significant cluster was found when compared these two groups during the single task (see Figure S5).

**Table S2.** Source comparison between fibromyalgia group and healthy control group during single and dual task.

| Cluster number | Location  (Tailarach coordinates) | Anatomical area |
| --- | --- | --- |
| Dual task | | |
| 2 | X= -57, Y= -9, Z= -1 | L Superior temporal gyrus |
| 5 | X= 67, Y= -9, Z= -7 | R Middle temporal gyrus |
| 7* | X= 11, Y= -73, Z= 15 | R Cuneus |
| 8 | X= 35, Y= 4, Z= 60 | R Middle frontal gyrus |
| 10* | X= 1, Y= 58, Z= -12 | R Medial frontal gyrus |
| Single task | | |
| 2 | X= 44, Y= -47, Z= 20 | R Superior temporal gyrus |
| 7 | X= -53, Y= -37, Z= 4 | L Middle temporal gyrus |
| 9* | X= 8, Y= 48, Z= 7 | R Medial frontal gyrus |

R: Right; L: Left.

During the dual-task, significant differences were found in the ERPS, mainly located between 5-9 Hz (see Figure S3 and Figure S4) when compared fibromyalgia and control group. The centroid dipoles were located in the right cuneus (Talairach coordinates X= 35, Y= 4, Z= 60) (Figure S3) and the right medial frontal gyrus (Talairach coordinates X= 1, Y= 58, Z= -12) (Figure S4).


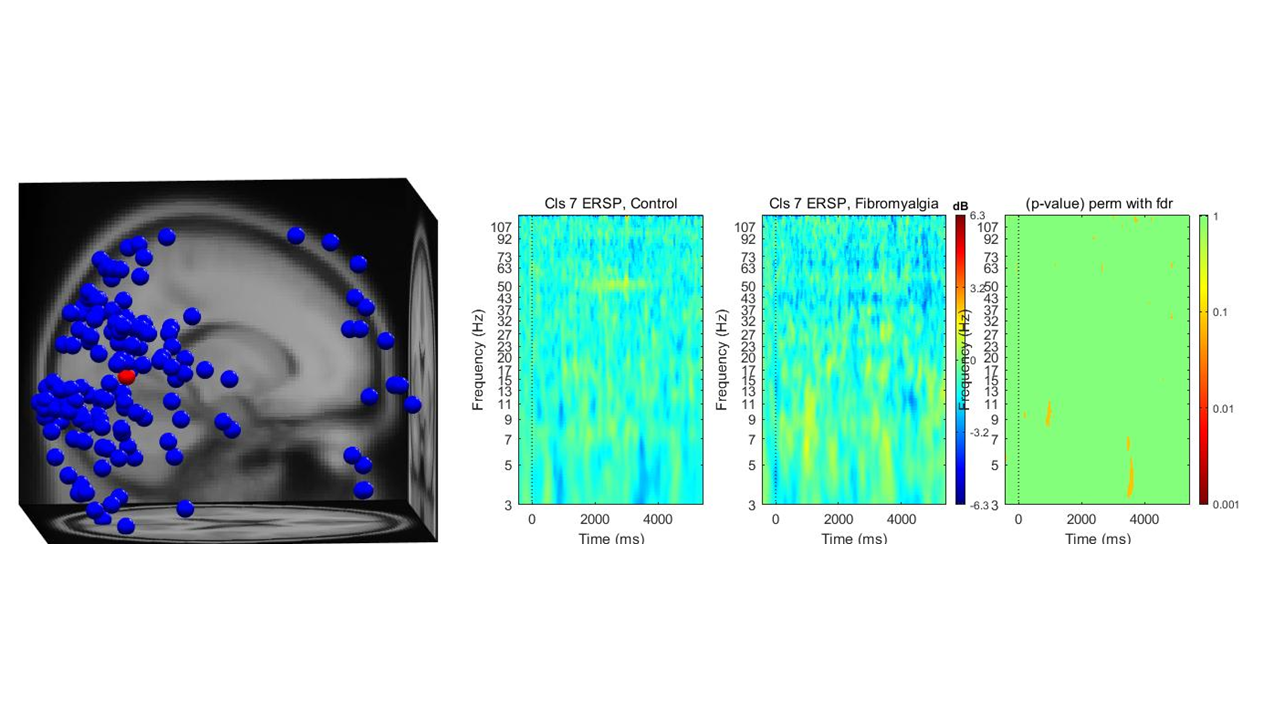


**Figure S3.** Dipoles location and ERSP of a cluster of dipoles when compared fibromyalgia and healthy control group during the dual-task condition.


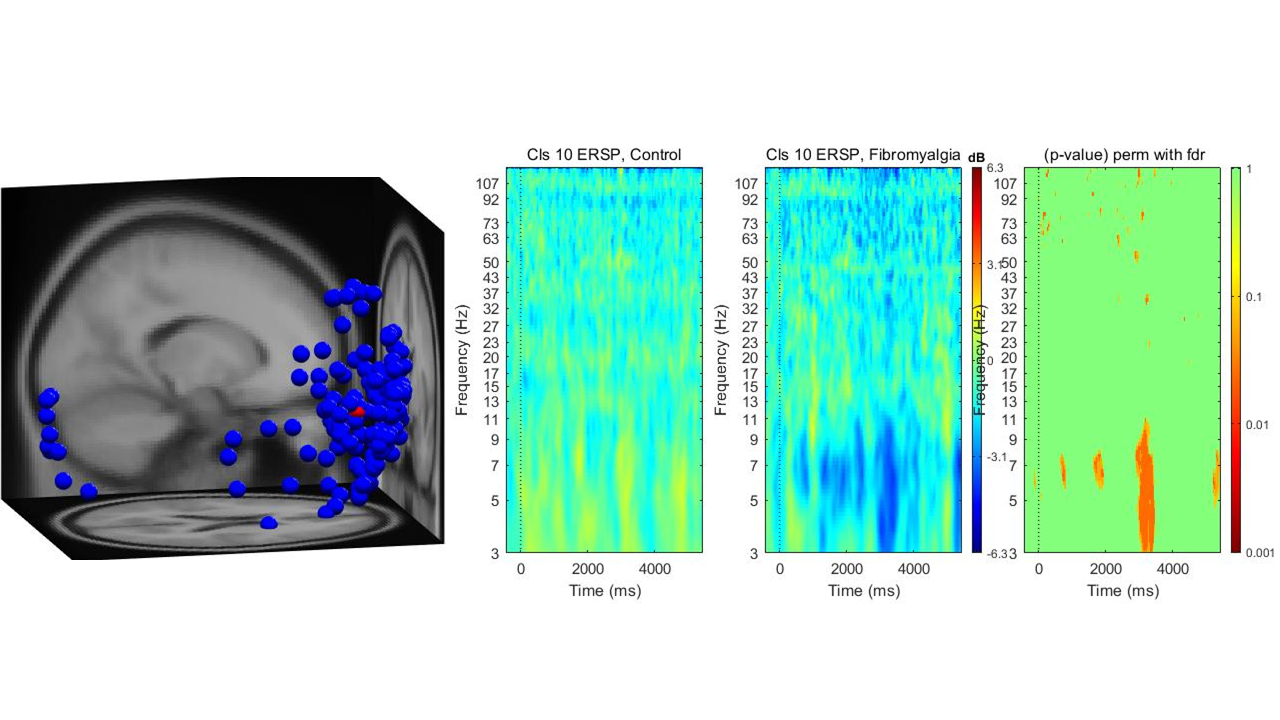


**Figure S4.** Dipoles location and ERSP of a cluster of dipoles when compared fibromyalgia and healthy control group during the dual-task condition.

During the single, significant differences were found in the ERPS, mainly located between 5-7 Hz (see Figure S5) when compared fibromyalgia and control group. The centroid dipole was located in the right medial frontal gyrus (Talairach coordinates X= 8, Y= 48, Z= 7).


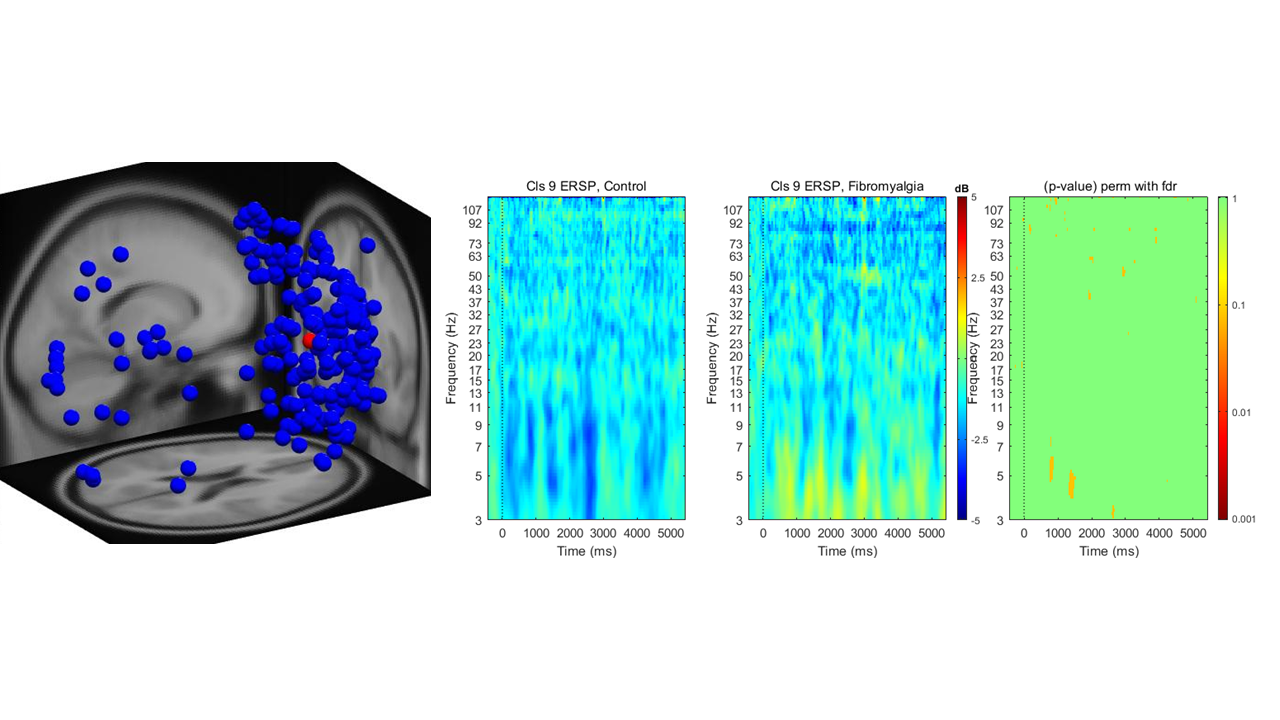


**Figure S5.** Dipoles location and ERSP of a cluster of dipoles when compared fibromyalgia and healthy control group during the single-task condition.
